# The influence of the cage system and colonisation of *Salmonella *Enteritidis on the microbial gut flora of laying hens studied by T-RFLP and 454 pyrosequencing

**DOI:** 10.1186/1471-2180-11-187

**Published:** 2011-08-22

**Authors:** Steen Nordentoft, Lars Mølbak, Lotte Bjerrum, Jantina De Vylder, Filip Van Immerseel, Karl Pedersen

**Affiliations:** 1 National Veterinary Institute, Technical University of Denmark, Section for Poultry, Fish and Fur Animals, Hangovej 2, DK-8200 Aarhus N, Denmark; 2 National Veterinary Institute, Technical University of Denmark, Bülowsvej 27, DK-1790 V, Denmark; 3 Danish Technological Institute, Kongsvang Allé 29, DK-8000 Aarhus C, Denmark; 4 Department of Pathology, Bacteriology and Avian Diseases, Faculty of Veterinary Medicine, Ghent, University, Salisburylaan 133, B-9820 Merelbeke, Belgium; 5 National Food Institute, Technical University of Denmark, Bülowsvej 27, DK-1790 V, Denmark

## Abstract

**Background:**

In the EU conventional cages for laying hens are forbidden beginning in January 2012, however concerns about a higher transmission rate of *Salmonella *in alternative cages systems have been raised. The extent to which cage systems may affect the intestinal microbiota of laying hens is not known, and different microbiota may demonstrate different resistance towards colonization with *Salmonella*. To investigate this, ileal and caecal samples from two experimental studies where laying hens were inoculated with *Salmonella *Enteritidis and housed in different systems (conventional cage, furnished cage or aviary), were compared using Terminal Restriction Fragment Length Polymorphism (T-RFLP). The distribution of genera in the microbiota in caecum was furthermore described by next generation sequencing of 16S rDNA libraries.

**Results:**

Hens in the same cage type developed similar T-RFLP fingerprints of the ileal and caecal microbiota, and these could be separated from layers in the other cages types. No significant difference in the fingerprint profiles could be observed between *Salmonella *positive and negative samples from same cage. By deep sequencing of 16S rDNA libraries from caecum, 197 different Operational Taxonomic Units (OTU) were identified, and 195 and 196 OTU respectively, were found in hens in aviary and furnished cages, but only 178 OTU of these were recovered from conventional cages. The ratio between the dominating phyla or families and genera in the microbiota remained fairly constant throughout the study. *Faecalibacterium *and *Butyricimonas *were the most prevalent genera found in the caecal microbiota of layers irrespective of the cage type.

**Conclusions:**

Hens confined in the same cage group tend to develop similar microbiota in their ileum and caecum possibly due to isolation, while differences in the microbiota between cages may be caused by environmental or individual bird factors. Although the cages type had influence on composition of the microbiota in the layers by promoting higher diversity in furnished and aviary systems, we did not observe differences in colonization and excretion pattern of *Salmonella *from these groups. We suggest, that differences in group size and exposure to a more faecally contaminated environment in the alternative systems may explain the observed differences in diversity of the caecal microbiota.

## Background

Due to animal welfare considerations the EU has banned the use of conventional cages (CC) for laying hens from 2012, and alternative systems such as furnished cage systems (FC), floor systems or aviaries (AV) have been proposed to replace these [1]. Traditionally, hens have been housed in minor cages with groups of 4-6 individuals, and the alternative systems are based on larger groups of more than 60 hens. In these cages layers are provided more space and facilities for natural behaviour, however a more aggressive nature among the laying hens has been observed [2], and environmental problems with a higher bacterial contamination level have also been noted [1]. This has led to concerns about an increased risk of transmission of *Salmonella *to humans due to a general higher level of microbial contamination of the shell of eggs derived from hens housed in alternative housing systems [3]. It is not known whether the combination of larger group sizes and social stress may increase the susceptibility to colonization by *Salmonella*. Stressing laying hens by feed withdrawal is a traditional method to induce molting, and in several studies this have resulted in an increase in the susceptibility towards colonization by *Salmonella *[4,5]. The mechanism behind this is not well understood, but the starvation may affect the balance between different microbial populations in the intestinal microbiota [5-7], as a reduction in diversity is observed which may lower the natural competitive barrier [5].

In the investigations of the dynamics in the intestinal microbiota, molecular methods have superseded conventional culture methods due to an increased sensitivity, and have become powerful tools. Terminal Restriction Fragment Length Polymorphism (T-RFLP) and Denaturant Gradient Gel Electrophoresis (DGGE) have been used to describe variations and diversity of the microbiota in the intestinal tract in broilers [8-10]. However, when it comes elucidate the phylogenetic diversity in the intestinal microbiota at species level, these methods are not sensitive and specific enough. By traditional culture methods only culturable genera are detected, and these are estimated to be about 1% of all genera present in the microbiota [11], whereas DGGE only detects species that represent more than 1% of the total microbiota [12], and in T-RFLP, sequence redundancy at the cleaving side may generate fragments of the same length from various species. A more comprehensive description of the distribution of species in the microbiota can be done by Sanger sequencing of 16S rDNA libraries. With this method individual species are arranged into Operational Taxonomic Units (OTU) based on > 98% similarity of 16S rDNA sequences [8,13], but as these methods are very laborious, only the most dominating species are detected. A much deeper investigation of the microbiota has been achieved with the introduction of second generation sequencing technology, such as 454 pyrosequencing, where massive parallel sequencing of short hyper variable regions within the 16S rDNA is performed [14-16]. Using this technology, a 16S rDNA library may be sequenced in one run; generating a large number of sequence reads that allows a much deeper insight in the distribution of species. Although the generated sequences do not cover the whole gene, Huse et al. [17] were able to achieve a 99% correlation of identification, when compared with full length sequencing of a library from the human microbiota.

The microbiota of laying hens experiencing nutritional stress has been investigated by 454 pyrosequencing [5]. In this study, the authors described the changes in the microbiota induced by different molting methods, where hens were given different feed or being starved. By starving the layers, they observed a decrease in species diversity of the caecal microbiota which was not found in hens receiving a diet with high fiber content. With the change to more welfare friendly cage systems, laying hens are now going to be housed in larger groups of 60 birds, rather than 4-6 birds as seen in conventional battery cages. Whether these changes in group size, increased contact between individuals or change in behavior may also have influence on the diversity of the species in the intestinal tract or in the oviduct, have not been investigated. Using molecular methods as T-RFLP and next generation sequencing, the aim of this study was to describe the effect of the housing system on the distribution of the dominating bacterial species in the intestinal microbiota in laying hens housed in different cage systems before and after inoculation with *Salmonella *Enteritidis.

## Results

### T-RFLP analysis of the impact of cage type on intestinal microbiota

The microbiota in ileal and caecal samples from the first experiment were characterised by creating individual T-RFLP fingerprint profiles for each sample. Profiles were generated on the basis of the number of Terminal Restriction Fragments (T-RFs) in the range of 60 - 850 bp. The relationship between two profiles could then be calculated by pair wise comparisons as a Dice similarity coefficient (S_D_), however to compensate for the variation between individual comparisons, the mean of the S_D _values was calculated and used to compare cage groups. The Dice coefficients from the first experimental study are shown in Table 1. In ileum, the highest Dice score was found between samples within same cage, and especially CC and AV diverged clearly from each other (S_D _54.3 ± 9.6) with FC being in between, sharing profiles with both the other cages (CC S_D _67.4 ± 9.9 and AV 66.8 ± 11.4). When sampling was done 4 weeks later, higher S_D _values were calculated within cage, while values between cages were in the range 65.5-67.5. This shows that layers sharing the same environment also had comparable ileal microbiota, and this similarity increased over time. The height of the T-RF peaks reflected the prevalence of individual species in the microbiota. Ileum was characterized by having the same 3-4 dominating T-RFs in all cage groups, but other individual T-RFs were also present. Before inoculation 10.5 ± 1.7 different T-RFs were detected in CC, while FC had 6.5 ± 2.7 and AV 7.3 ± 3.5. These were maintained throughout the study, although an increase was found in AV (10.7 ± 2.7). The four most dominating T-RFs in all samples were 393 bp, 406 bp, 597 bp, and 550 bp. These T-RFLP fragments could be equated with by different *Lactobacillus *species by *in silico *digest of 16S rDNA. Although the total number of detectable T-RFs remained constant in the ileum, an inverted relationship was found between one group of T-RFs: 406 bp, 606 bp and 550 bp which decreased in height, whereas as a new and unidentified T-RF 813 bp emerged. This shift was primarily found in layers from FC and a few layers from other cages, and this may explain some of the differences observed in S_D _between cages.

**Table 1:** Comparisons of T-RFLP profiles of microbiota in the ileum and caecum of layers housed in different cage systems

Before Inoculation						
				Mean S_D_		
				
Location	Cage	n	T-RF	Conventional	Furnished	Aviary

Ileum	Conventional	4	10.5 ± 1.7	70.5 ± 12.4	-	-
	Furnished	4	6.5 ± 2.7	67.4 ± 9.9	65.9 ± 7.5	-
	Aviary	4	7.3 ± 3.5	54.3 ± 9.6	66.8 ± 11.4	72.3 ± 7.0
	
Caecum	Conventional	4	39.5 ± 6.6	66.4 ± 6.0	-	-
	Furnished	4	39.8 ± 4.2	60.8 ± 3.5	75.1 ± 6.0	-
	Aviary	4	52.7 ± 23.5	38.6 ± 6.3	38.5 ± 4.8	45.4 ± 14.5

**Four weeks PI**						

				Mean S_D_		
				
Location	Cage	n	T-RF	Conventional	Furnished	Aviary

Ileum	Conventional	8	10.0 ± 1.2	86.5 ± 10.1	-	-
	Furnished	8	6.9 ± 2.2	65.5 ± 9.3	81.1 ± 6.9	-
	Aviary	7	10.7 ± 2.7	66.8 ± 9.2	67.5 ± 9.2	73.8 ± 9.0
	
Caecum	Conventional	8	58.0 ± 5.2	73.4 ± 5.8	-	-
	Furnished	8	51.3 ± 7.3	57.7 ± 8.1	67.1 ± 8.6	-
	Aviary	8	63.6 ± 5.3	54.6 ± 4.7	58.2 ± 4.9	74.2 ± 4.9

n: number of samples

S_D_: Dice similarity coefficient

T-RF: Terminal Restriction Fragments

The T-RFLP profiles from the caecum contained a higher number of T-RFs reflecting a much more complex microbiota than in the ileum, and an increase in the amount of T-RFs was observed in all caecal microbiota over time (Table 1). The majority of the dominating T-RFs were shared by all cage groups, however cages specific differences among the minor T-RFs were observed. Samples from CC and FC were more uniform, whereas a large variation between the profiles was observed in AV on the first sampling day (S_D _45.4 ± 14), however the profiles were more uniform on the second sampling 4 weeks later (AV 74.2 ± 4.9). The S_D _values were higher within the same group than between cage groups, and an increase in S_D _over time was observed, in accordance with the findings from the ileum.

**Figure 1:**
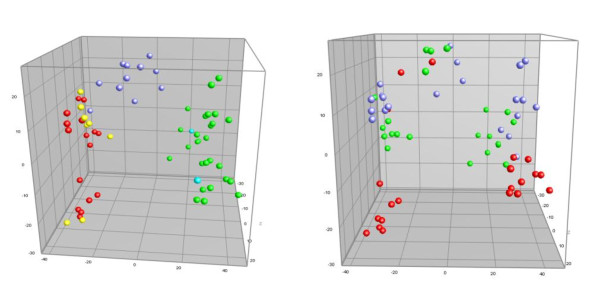
**PCA comparison of T-RFLP profiles of the caecal flora in laying hens housed in different cage systems**. PCA analysis of T-RFLP generated fingerprints of the bacterial community in caecal samples from 2 experimental studies. The first plot shows all samples from both experiments coloured according to sampling time and salmonella status. Samples collected before inoculation with *S*. Enteritidis (blue) were clearly separated from samples collected 4 weeks PI (red and yellow). The second experiment (green, light blue) was also clearly separated from the first experiment (X = 20.7%, Y = 10.1%, Z = 9.0%). Yellow and light blue represents samples positive for *Salmonella*. In the second plot, the same samples are marked according to cage system. Each cage type are separated in clusters with the major variance being 20.7% between experiments and Y = 10.7% between cages. Red dots: Aviary, Green dots: Conventional cage, Blue: Furnished cage.

To test whether the differences in profiles between cages were caused by a specific cage factor or merely a reflection of isolation between cages, we included samples from the second experimental study [18]. Apart from one T-RF (550 bp.), all dominating T-RFs in the ileum from the first trial were also present in a second study. The major groups of T-RFs in the caecal samples were similar between experiments; however some fragment were only found in one of the experiments. To test for common cage factors, profiles from the caecum were compared by Principal Component Analysis (PCA) (Figure 1). A clear clustering of samples from the same experiment and cage system was observed. By the first principal component (X = 20.7%) all caecal T-RFLP profiles were clearly separated in two groups according to sampling day and experiment, thus showing that the highest variance was caused by differences between the two experiments. The second component (Y = 10.1%) separated each experiment into three clusters each containing profiles from same cage system. In both studies CC samples were most different from AV, with FV samples clustering in between. Samples collected before inoculation did not cluster as clearly as samples taken at the end of the study. An indication of a common cage factor was observed by the Y component, where samples from the same cages in both experiments were influenced similarly by this component. The PCA showed that especially T-RF 393 was more prevalent in samples from CC, while T-RF 102 was more frequently found in AV. It is likely that the first fragment may represent a *Lactobacillus *spp., while no specific genera could be identified for the other fragment, as several different genera (*Bacteroides, Prevotella *or *Porphyromonas*) may be represented by this T-RF.

### T-RFLP analysis of the impact of *Salmonella *on the intestinal microbiota

The impact of an inoculation with *S*. Enteritidis on intestinal microbiota was also evaluated. After inoculation, no clinical signs of infection were detected in the layers. However, colonisation of the intestinal microbiota was established, and *S*. Enteritidis could be detected in samples from internal organs as well as in cloacal swabs [18,19]. At the end of both studies, *Salmonella *was found in a few layers by culture and PCR. In the ileal samples, *Salmonella *was detected in 2/8 from AV by PCR, while other samples were negative. In the caecum, *S*. Enteritidis could be cultured in 2/8 samples from AV, 3/8 from both FC and CC. The concentration of *S*. Enteritidis in the positive samples was generally low, as culture positive samples not always were positive by real-time PCR. T-RFLP profiles of intestinal microbiota positive for *S*. Enteritidis were compared with profiles where it had been eliminated. On the basis of the mean SD values calculated between *Salmonella *negative and positive samples from the same cage, no differences could be detected between positive and negative samples within same cage (data not shown). When profiles were analysed by PCA, no discrimination was found between positive or negative samples within the same cages (Figure 1).

### 454 sequencing of the caecal microbiota

The microbiota in the caecal samples from the first experiment were further characterized by 454 pyrosequencing of 16S rDNA gene libraries. Due to the high sample similarity observed in the T-RFLP analysis, we pooled the DNA from 10 cage mates and used this as template for 454 pyrosequencing. In total six samples were generated, one for each cage type before and after inoculation with *Salmonella*. From each sample, between 20,000 and 50,000 sequence reads could be used for analysis (Table 2). On the basis of 99% similarity these reads were sorted into OTUs. The amount of reads in each OTU varied greatly ranging from more than 14,000 to single reads, thus reflecting the prevalence in the microbiota. Due to differences in the amount of sequence reads obtained from individual samples, the relative distribution of sequences was calculated on the basis of the total number of reads from the sample. OTUs that accounted for > 1% of the total number of sequences were considered as dominant species.

**Table 2:** The distribution of sequence reads, OTU's in absolute numbers and the ratio between *Firmicutes *and *Bacteroides *in pooled caecal samples

	Conventional cage		Furnished cage		Aviary	
	
	Before inoculation	4 weeks PI	Before inoculation	4 weeks PI	Before inoculation	4 weeks PI
Number of reads	51,863	21,714	42,885	42,520	51,715	40,410

Number of OTU/total number of OTU	185/197	178/197	196/197	193/197	195/197	193/197
	93.9%	90.4%	99.5%	98.0%	99.0%	98.0%

Firmicutes/Bacteroides ratio^a^	0.81	0.61	0.87	0.74	0.69	0.68

^a ^The ratio was calculated by dividing all OTU that could be affiliated to *Firmicutes (Clostridia *and *Bacilli*) by the number of OTU's from *Bacteroides*.

**Figure 2:**
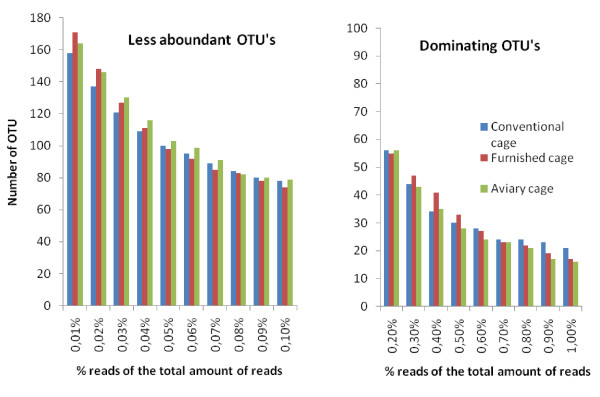
**The distribution of OTU's according to the prevalence in the microbiota**. The number and prevalence of OTU based on the relative prevalence in each sample (cut off < 0.01%). The number of different OTU's in the group of less abundant genera was highest in furnished and aviary cage, in contrast to conventional cage where we observed fewer but more dominating genera.

In total, 197 different OTUs were identified, and 196 and 195, respectively, out of these were found in non-inoculated samples from AV and FC, however, for CC a progressive decrease in numbers of OTUs was observed in both samples before and after inoculation with *Salmonella*. In these cages, 185 OTUs were identified before inoculation and 178 OTUs four weeks after inoculation, while in the other cages 193 OTUs were detected at the end of the experiment. Due to a different number of reads obtained from each sample, normalized prevalence values of each OTU were calculated. Using a cut-off value of 0.01%, the difference in diversity between cages was still observed where the dominating genera in CC constituted a larger proportion of the microbiota at the expense of fewer OTU's, compared to the two other cages (Figure 2).

**Figure 3:**
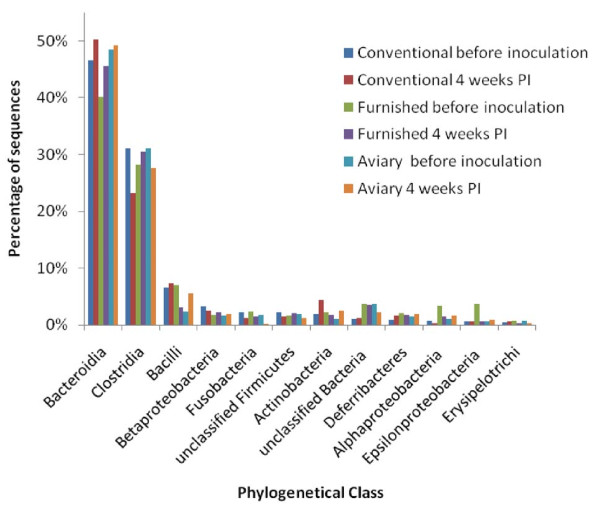
**Taxonomic distribution of bacterial classes in caecum**. Taxonomic distribution of bacterial classes in caecum from layers caged in different cage systems. The distribution is based on pyrosequencing of 16S rDNA libraries generated from pooled caecal samples. Samples were taken before inoculation with *S*. Enteritidis and 4 weeks PI.

The consensus sequence from each OTU was compared against the Ribosomal Database (RDP server) to find the most related species or genus. Though many of the bacterial species in the caecal microbiota still remain to be characterized, it was possible to classify 92% of all OTUs to phylum level, and out of these were 86% classified to class level and 55% to genus level. Although variation was observed in the relative presence that colonized the caecum, it was the same group of genera that were dominating in all cages before and after inoculation, accounting for more than 74% of the total amount of reads (Table 3). The caecal microbiota from all cages was dominated by two phyla: *Bacteroidetes *and *Firmicutes*, with *Proteobacteria, Fusobacteria, Actinobacteria *and *Deferribacteres *also being represented, but only in low numbers. The dominating classes were *Bacteroidia *and *Clostridia *(Figure 3), and within these classes were *Butyricimonas *spp. and *Faecalibacterium *spp. the most dominating genera. Based on identified OTU's, a ratio between *Bacteroidetes *and *Firmicutes *was calculated (Table 2). All samples had a relatively high number of *Bacteroidetes*, with the exception of CC where a drop was observed in samples collected 4 weeks post infection (PI).

**Table 3:** Listing of the most prevalent genera in caecal samples accounting for more than 1% of sequence in one or more samples

			Conventional		Furnished		Aviary	
Class	Family	Genus	Before inoculation (%)^a^	4 Weeks PI (%)	Before inoculation (%)	4 Weeks PI (%)	Before inoculation (%)	4 Weeks PI (%)
*Bacteroidia*	*Rikenellaceae*	*Alistipes*	2.3	1.1	1.4	1.7	1.4	1.2
	*Bacteroides*	*Bacteroides*	1.4	1.4	5.3	4.8	6.2	5.6
	*Bacteroidaceae*	*Bacteroides*	2.1	2.5	0.7	2.1	1.7	2.6
	*Porphyromonadaceae*	*Barnesiella*	1.2	3.1	1.1	2.0	2.3	1.4
	*Porphyromonadaceae*	*Butyricimonas*	28.8	20.6	12.4	14.7	13.8	18.8
	*Porphyromonadaceae*	*Parabacteroides*	2.8	4.4	4.9	5.4	4.6	3.8
		Unclas. *Bacteroidales*	4.4	9.8	9.0	7.1	10.3	8.9
		Unclas. *Bacteroidales*	0.7	2.6	2.1	3.0	4.9	2.5
		Unclas. *Bacteroidales*	0.2	2.0	1.0	2.5	1.0	1.9
		**total for class**	**43.8**	**47.4**	**37.8**	**43.1**	**46.2**	**46.6**

*Clostridia*	*Clostridiales*	*Blautia*	0.6	0.4	1.3	0.5	1.1	0.4
	*Ruminococcaceae*	*Faecalibacterium*	18.6	11.6	13.6	19.0	16.7	13.9
	*Veillonellaceae*	*Phascolarctobacterium*	4.3	0.9	2.6	0.4	1.8	3.8
	*Ruminococcaceae*	*Subdoligranulum*	0.0	0.2	1.4	1.6	0.9	0.4
		**total for class**	**23.6**	**21.0**	**19.0**	**22.0**	**20.0**	**23.0**

*Bacilli*	*Lactobacillaceae*	*Lactobacillus*	3.8	0.4	0.3	0.1	0.2	0.1
	*Lactobacillaceae*	*Lactobacillus*	2.3	4.8	5.4	2.5	1.9	5.0
		**total for class**	**6.1**	**5.3**	**5.7**	**2.5**	**2.1**	**5.1**

*Betaproteobacteria*	*Alcaligenaceae*	*Sutterella*	1.5	1.1	0.5	0.7	0.3	0.6
	*Alcaligenaceae*	*Sutterella*	1.1	0.8	0.6	0.9	0.6	0.8
		**total for class**	**2.6**	**1.9**	**1.1**	**1.6**	**0.9**	**1.4**

*Fusobacteria*	*Fusobacteriaceae*	*Fusobacterium*	2.2	1.3	2.4	1.5	1.8	0.1
*Actinobacteria*	*Coriobacteriaceae*	*Olsenella*	1.7	3.9	1.8	1.4	0.4	1.9
*Deferribacteres*	*Deferribacteraceae*	*Mucispirillum*	0.9	1.7	2.0	1.8	1.5	1.9
*Epsilonproteobacteria*	*Helicobacteraceae*	*Helicobacter*	0.5	0.6	3.6	0.6	0.5	0.8
*Synergistia*	*Synergistaceae*	*Cloacibacillus*	0.9	2.0	1.1	1.0	1.3	1.0
*Alphaproteobacteria*		Unclas. *Alphaproteobacteria*	0.2	0.1	1.6	0.5	0.3	0.4
Unclas. *Bacteria*		Unclas. *Bacteria*	0.2	0.6	2.6	2.0	2.0	1.5
Unclas. *Firmicutes*		Unclas. *Firmicutes*	1.1	0.7	0.6	1.1	1.1	0.6

^a) ^Percentages are presented as the relative distribution compared to all OTU's in the sample.

## Discussion

To investigate the impact of different housing conditions on the intestinal microbiota in ileum and caecum in laying hens, samples obtained from two experimental inoculation studies previously reported by De Vylder et al. [18,19] have been further characterised using T-RFLP and 454 pyrosequencing. We found that individuals living in the same environment also tend to develop similar microbiota. Despite of being raised in the same environment and likely having similar microbiota to begin with, we found, that when hens were transferred to different cages types (conventional cages, furnished cages or aviary) for 2 weeks, minor but uniform changes in the T-RFLP profiles of the microbiota in ileum and caecum occurred. By comparing T-RFLP fingerprints from individual hens, we found highly similar ileal and caecal profiles in hens from same cage, which could be discriminated from other cages in the same experiment. However, the differences were not cage type specific, as when samples from two independent experiments were compared by PCA, the largest component were observed between experiments, meaning that cage type only had minor influence on the variance. This indicates that the intestinal microbiota may be influenced on the contact to the surrounding microbiological environment in the cage.

The differences in the evolution of the microbiota were further analysed by deep sequencing of 16S rDNA libraries from pooled caecal samples. When 16 week old laying hens were moved from a floor system and into conventional cages, their caecal microbiota changed towards a less diverse microbiota compared to hens from the same flock that were allocated to aviary and furnished cages. Sequencing of rDNA libraries revealed that hens housed in conventional cages showed a progressive decrease in the number of different OTUs in their caecal microbiota, compared to hens housed in aviary or furnished cages. The decline was already observed after 2 weeks in the cage, and it was even more pronounced after 4 weeks. The same reduction was not observed in the other cage systems. The OTUs that were not recovered in conventional cages were all represented in the other cages, however in low numbers reflecting that they belong to the group of less abundant species. As each OTU represents unique genera or even species, this reflects an overall decrease in diversity of their caecal microbiota towards fewer and more dominating species. Alternative cage systems are characterized by having larger cages due to flock sizes and facilities for enhancing natural behaviour. These facilities may, however, hinder the removal of manure compared to conventional cages, and an overall higher bacterial level has been noted in these systems [1]. It is likely that the laying hens housed in a more contaminated environment, as in the alternative systems, may be more exposed to faeces from the other layers, and thereby continuously being reinoculated, thereby maintaining a higher species variety in the microbiota.

The vast amount of sequence reads generated from each sample by 454 pyrosequencing allowed quantification of the individual OTU in relation to the total flora. Only minor differences were observed in the relative distribution of phyla and classes of bacteria in the caecal microbiota between cages, but quantitative variations that were not cage specific were observed between different genera. However, when OTUs were grouped according to phyla and classes, comparable groups were found in all samples. This indicates that the cage system itself did not influence the balance between the large classes, but pinpoints the caecal microbiota as a dynamic, highly competitive organ where a decrease in one genus may be compensated by an increase in a closely related species, or other species belonging to the same functional guild that shares the same requirement for substrates.

When the consensus sequences from 197 OTUs were aligned with the RDP database, more than 91% were identifiable at least to phylum level, and more than 55% could be identified to genus level. The most prevalent phyla in the caecal microbiota were *Bacteroidetes*, with *Firmicutes *being the second most prevalent. The ratios between these two phyla (F/B) remained fairly equal between the CC and AC, but a decrease was observed for CC. A major reason for this difference was promoted by a shift from *Faecalibacterium *to *Butyricimonas*. Whether this change was mediated by the cage system of a coincidence remains to be established, but we did not find that it changed the susceptibility for *Salmonella*, probably because both species produces butyric acid. There are indications that the feed may have large influence the F/B ratio. In domestic and wild turkeys, Scupham et al. [20] found similar ratios between these phyla; however this is in contrast to the caecal microbiota found in broilers. In a number of studies [8,13,21,22], the microbiota in broilers were heavily dominated by *Firmicutes*, with *Bacteroidetes *only present at much lower level. An explanation for this may be the different feeding strategies that are used. Broilers are normally fed a high energy diet that sustains fast growth, which possibly leaves more digestible nutrients for the intestinal microbiota. In contrast, laying hens are fed a much more restricted diet containing less energy and higher amounts of digestive fibers, which instead may favour genera from *Bacteroidetes*. The same phenomena has been described for the microbiota in obese humans, where Ley et al. [23] observed an increase in *Bacteroidetes *during long term restricted diet.

The two most dominating genera found in this study were *Faecalibacterium *and *Butyricimonas *constituting more than one third of the total microbiota in all sequenced caecal samples. The first species is a well known colonizer of the caecal microbiota of poultry; however *Butyricimonas *has just recently been described in rats [24], and has to our knowledge not been described in poultry before. Both bacteria are important contributors to the production of butyric acid in the lower intestine, and may represent an important functional guild in the microbiota. Studies have shown that especially butyric acid may have a prominent role in the reduction of invasion [25], and colonization of *Salmonella *in the caecal microbiota [26]. *Butyricimonas *was the most dominant genus in caecum samples from conventional cages, but this difference was not reflected in any variations found in the colonization level of *S*. Enteritidis as reported by De Vylder et al. [19], who found no difference in excretion level and time between cage systems.

We did not find evidence that the introduction of *S*. Enteritidis to the intestinal microbiota were able to change the species diversity in ileum or caecum. When individual T-RFLP profiles from *Salmonella *positive layers were compared with cage mates that had cleared the infection no differences were observed. When comparing the distribution of OTU in each group before and after inoculation, the balance between different classes and genera were also maintained throughout the study. The low impact on the intestinal microbiota may be explained by the fact that inoculation only induced a subclinical infection, in contrast to experimental studies where a more profound disturbance of the microbiota has been observed in cases where diarrhoea has followed infection [27,28].

In the early studies of Nurmi and Rantala [29], it was shown that a highly diverse intestinal microbiota in broilers is one of the best barriers towards colonization with *Salmonella *(competitive exclusion). However, we did not find that decreased diversity in the layers had a significant impact on the colonization and elimination of *Salmonella*. It is likely that this colonisation resistance is highly important in broilers where a mature flora has not been established yet, but in layers this may not be as important. Furthermore, in the second inoculation study where seeder birds were housed together with non-infected birds, De Vylder et al. [18] found that the transmission of *S*. Enteritidis was higher among hens housed in aviary or floor system than in conventional and furnished cages. A likely explanation for our observation is that direct contact to faecal material from infected hens is very important for the transmission of *S*. Enteritidis in a flock, and that the higher species diversity found in layers with more contact with faecal material does not prevent colonization, but keeps it at a relatively low level.

## Conclusions

In the present study, we have compared the intestinal microbiota in layers from different housing systems under experimental conditions. When laying hens were housed in conventional cages, a change was observed in their caecal microbiota towards a less diverse flora, with the most prevalent genera being more dominating compared to aviary and furnished cage. This decrease in diversity did not have an impact on the elimination of *Salmonella*, and moreover the higher diversity found in aviary systems did not protect laying hens from being infected. We did not find evidence, that the cage systems itself was able to change the intestinal microbiota in a way which made it more sensible towards colonization with *Salmonella*, but it highlights that hygiene in alternative systems is a particularly critical factor for preventing the spread of *Salmonella *within a flock.

## Methods

### Samples for analysis

Intestinal content samples from ileum and caecum were received from two experimental infection studies previously described by De Vylder et al. [18,19]. Briefly, in the first experiment 16 week old laying hens raised in a floor systems, were allocated into three different cage conditions (conventional, furnished and aviary cage system). After 2 weeks of accommodation were all hens inoculated with 1.5 × 10^8 ^CFU of a nalidixic acid resistant *S*. Enteritidis PT 4 strain (76Sa88), which previously had been isolated from an outbreak of salmonellosis in laying hens [30] chain fatty acid). The development of the infection was followed by conventional culture methods until the slaughter 4 weeks later. Samples for microbiota composition analysis were collected prior to inoculation (Week 18) and at the 4 weeks (Week 22) post infection (PI).

In the second experiment 16 week old laying hens raised in a floor systems, were accommodated for two weeks in one isolation unit (floor system) to adjust to their new environment. Then the flock was randomly divided in two groups, and one hundred and twenty-six non-inoculated contact animals were housed in 3 different housing systems; (1) 36 hens in battery cages, (2) 30 hens in a furnished cage, (3) 30 hens in an aviary. The remaining one hundred and twenty-six hens, called seeder-hens, stayed on the floor and were individually inoculated orally with 10^9 ^CFU of the same nalidixic acid resistant *Salmonella *Enteritidis strain. At day 22 post-infection, the seeder hens were randomly divided into four groups and housed together with the non-infected contact hens in the different housing systems such that in each housing system fifty percent seeders and fifty percent contact animals were present. Samples of ileal and caecal content were collected for analysis of the microbiota at the end of the experiment 4 weeks later. Al experiments were approved by the Ethical Committee of the Faculty of Veterinary Medicine, Ghent University.

### Extraction of DNA

During necropsy of layers, samples were collected from the ileum and caecum. The gut samples were stored by diluting 1 g with 3 ml of 98% ethanol and kept at 4°C until purification, where the ethanol was removed by washing twice with 1 ml of Buffered Peptone Water (Oxoid, Basingstoke, UK). Oviduct samples were stored at -20°C until preparation, where surface samples from these organs were collected by scraping the mucosal lining after gentle thawing. Two hundred milligrams of gut contents (ileum and caecum) or oviduct were used for total DNA extraction using the QIAamp DNA Stool Mini Kit (Qiagen, Hilden, Germany) system. The extraction was carried out in accordance with the instructions of the manufacturer, with an additional step of lysozyme treatment, which was added to the procedure before the use of InhibitEX tablets provided in the QIAamp DNA Stool Mini Kit. After incubation of the sample in ASL buffer at 95°C for 5 min, 140 μL of a 10 mg/ml solution of lysozyme (Sigma-Aldrich, Brøndby, Denmark) in Tris-EDTA buffer (10:1 mM), pH 8, was added to each extraction tube and samples were incubated at 37°C for 30 min. The purified DNA was eluted in 200 ml buffer AE (Qiagen) and DNA was stabilized by adding 4 μL of a 50 mg/ml BSA solution (Ultrapure BSA, Ambion, Applied Biosystems, Naerum, Denmark, cat. no. 2616) and 2 μL of Ribonuclease-A (Sigma-Aldrich, R-4642). The purity and concentration of DNA was measured using NanoDrop (NanoDrop Technologies, Wilmington, Delaware, USA). All samples were stored as concentrated samples at -20°C until use. Samples were diluted to a concentration of 5 mg DNA per ml before use.

### Real-time PCR for the detection of *Salmonella*

Extracted total DNA samples from the ileum and caecum were tested for *Salmonella *by a LNA real-time PCR method described by Josefsen et al. [31] with minor modifications. PCR was performed on a MX3005P (Stratagene, La Jolla, California) in a total reaction volume of 25 μl, consisting of 12.5 μl of Promega PCR Mastermix (Promega, Wisconsin, MA), 4.25 μl of water, 3 mM MgCl2, 1 mg/ml BSA (Sigma-Aldrich, cat L4390), 10 pmole of forward primer ttr-6 (5'-CTCACCAGGAGATTACAACATGG-3'), 10 pmole of reverse primer ttr-4 (5'-AGCTCAGACCAAAAGTGACCATC-3'), 10 pmole of LNA target probe (6-FAM-CG+ACGGCG+AG+ACCG-BHQ1) (Sigma-Aldrich) and 2 μl of purified DNA (10 ng). The temperature profile was initial denaturation at 95°C for 3 min., followed by 40 cycles of 95°C for 30 s, 65°C for 60 s, and 72°C for 30 s. Fluorescence measurements were analyzed with the MxPro-Mx3005P software (Stratagene, version 4.10). The threshold was assigned by using the software option background-based threshold. All samples were tested in duplicate and a sample was counted as positive if at least one out of two were positive.

### Polymerase chain reaction conditions for 16S rDNA

Generation of a PCR fragment of the 16S ribosomal gene was done as described previously [27]. Briefly, four replicate 50 μl PCR mixtures were made from each sample on a PTC-200 thermal cycler (MJ Research, Watertown, Massachusetts). Reaction conditions were as follows: 5 μl PCR buffer (HT Biotechnology Ltd., Cambridge, UK); 10 mM (each) deoxynucleoside triphosphates, 10 pmole forward primer S-D-Bact-0008-a-S-20 (5'-AGAGTTTGATCMTGGCTCAG-3'), 10 pmole reverse primer S-D-Bact-0926-a-A-20 (5'-CCGTCAATTCCTTTRAGTTT-3'), and 1.25 U of DNA polymerase (SuperTaq; HT Biotechnology Ltd., Cambridge, UK) in a 50- μl reaction. Primer S-D-Bact-0008-a-S-20 was 5' FAM labelled. Two-microliter aliquots of extracted DNA (10 ng) were used for amplification of caecal samples, but ileum samples were analysed using non diluted samples due to low DNA concentration. PCR cycling consisted of an initial denaturation at 94°C for 6 min; followed by 30 cycles of denaturation at 94°C for 30 s, annealing at 57°C for 45 s, and extension at 72°C for 2 min; and a final extension at 72°C for 3 min. Amplified DNA was verified by electrophoresis on 2% agarose gels.

### Restriction digest

The PCR products from the four replicates were pooled into two samples, purified with QIAquick PCR purification kit (Qiagen, Hilden, Germany), and finally eluted in a volume of 30 μl EB buffer (10 mM Tris, pH 8.5). Then 15 μl purified PCR product was digested overnight (or 3 hours) at 37°C with 0.02 U of *Hha1 *(Boehringer, Mannheim, Germany) in a 20 μl reaction mixture.

### Terminal-restriction fragment length polymorphism

Each sample was analysed as two replicate fragments (T-RFs) by electrophoresis on an automatic sequence analyzer (ABI-PRISM-373-DNA-Sequencer; PE Biosystems, Foster City, California). Aliquots (2 μl) of T-RFs were mixed with 2 μl of deionized formamide, 0.4 μl of loading buffer (PE Biosystems), and 0.6 μl of DNA fragment length standard (MegaBace ET900, GE Healthcare, Hillerød, DK). The T-RF mixture was denatured at 94°C for 2 min and chilled on ice prior to electrophoresis. Five microliter aliquots of the mixture were loaded on a 36-cm, 6% denaturing polyacrylamide gel. Electrophoresis settings were 2,500 V and 40 mA for 10 h, using the B filter set. Due to sequence species specific variations in the ribosomal gene, a restriction digest will give rise to T-RF of different size, and when many species are mixed as in the intestinal microbiota this can be visualized as a pattern of peaks in an electropherogram, a fingerprint profile. These profiles were collected by the software and analysed by the use of BioNumerics software (Applied Maths, Sint-Martens-Latem, Belgium). The length of each band was determined by comparing it towards the internal standard ladder. From each sample two replicates were compared, and weak bands that were only represented in one of the two were rejected to exclude false T-RFs from the fingerprint. After normalization of all profiles towards the internal standard, they were compared using BioNumerics. The comparisons between cages were based on calculating the Dice similarity coefficient and the unweighted pair group method using arithmetic averages for clustering. Principal Component Analysis (PCA) was performed to reflect the grouping and relatedness of samples.

### Pyrosequencing of ribosomal genes

Samples (n = 10) from the same cage types (CC, FC, and AV), and sampling date (before inoculation and 4 weeks PI.), were pooled by mixing 250 ng of purified DNA from each sample in one tube, in total making up 6 samples. After adding twice the volume of 70% ethanol to each sample for conservation, they were submitted for pyrosequencing at LGC Genomics, Berlin, Germany, using the Genome Sequencer FLX Titanium system's standard amplicon sequencing protocols (Roche Ltd, Basel, Switzerland). There, a 410-420 bp fragment spanning two variable regions (V4 and V5) in 16s rDNA genes was amplified using the primers 519F 5'-CAGCAGCCGCGGTAATAC-3 and 926R 5'-CCGTCAATTCCTTTGAGTTT-3, targeting *Bacteria*. To increase the number of reads, all samples were run as multiplex on the same ¼ picoplate using nucleotide barcodes tags on primers, allowing sample identification to each sequence read.

### Analysis of data from pyrosequencing

All sequences in the output file from the FLX sequencer was sorted into sample groups based on the barcode tag. After trimming all sequences for barcodes and fusion primers using the FLS software, sequences were imported into the CLC bio software (CLC bio, Aarhus, Denmark), where they were checked, aligned and filtered for high quality sequences. OTU's were generated by CLC based on 99% similarity on the data set that had a sequence longer than 400 bp. The Sequence match analysis tool in the Ribosomal database project 10 http://rdp.cme.msu.edu/ was used to assign the phylogenetic position of each OTU. The search criteria were for both type and non-type strains, both environmental (uncultured) sequences and isolates, near-full-length sequences (> 1200 bases) of good quality. If there was a consensus at the genus level, the tag was assigned this taxonomic classification. If no such consensus was found, the classification proceeded up one level to family, and again if no taxonomic affiliation could be assigned the tag continued to be proceeded up the tree, as described by Huse et al. [32]. In some cases, it was not possible to assign a domain, and these sequences might represent new organisms or the sequences might be biased; in these cases the tags were excluded from the dataset. In total 250,007 sequences were finally assigned a taxonomic classification in this study.

## Authors' contributions

SN carried out the molecular work, did the the statistical analysis of T-RFLP and wrote the manuscript. LM preformed sequence alignment and analysis of the pyrosequencing data. LB participated in the design of the molecular study. JDV and FVI designed and conducted the experimental studies, and KP conceived of the study. All authors read and approved the final manuscript. The authors declare that they have no competing interests.
